# Immunotherapeutic Approaches to Head and Neck Squamous Cell Carcinoma: Current Clinical Practice and Future Directions

**DOI:** 10.1002/hed.70385

**Published:** 2026-07-09

**Authors:** Carol R. Bradford, Sreeram M. P., Karthik N. Rao, Orlando Guntinas‐Lichius, Nabil Saba, Alfio Ferlito

**Affiliations:** ^1^ Department of Otolaryngology—Head and Neck Surgery University of Minnesota Medical School Minneapolis Minnesota USA; ^2^ Sri Shankara Cancer Hospital and Research Centre Bangalore India; ^3^ Head and Neck Surgical Oncology Department Sri Shankara Cancer Hospital and Research Centre Bangalore India; ^4^ ENT Department Jena University Hospital Jena Germany; ^5^ Department of Hematology and Medical Oncology The Winship Cancer Institute of Emory University Atlanta Georgia USA; ^6^ International Head and Neck Scientific Group Padua Italy

## Abstract

**Background:**

Head and neck squamous cell carcinoma (HNSCC) is frequently diagnosed at advanced stages and has historically been associated with poor outcomes. Immune checkpoint inhibition has transformed the therapeutic landscape, offering durable responses in recurrent, metastatic, and locally advanced disease.

**Methods:**

This review summarizes pivotal clinical trials evaluating immunotherapeutic strategies in HNSCC, including PD‐1/PD‐L1 inhibitors, combination regimens, and emerging modalities across recurrent/metastatic and curative‐intent settings, with attention to efficacy, safety, and biomarkers.

**Results:**

Phase III trials have established pembrolizumab and nivolumab as standards of care in recurrent or metastatic HNSCC, demonstrating improved overall survival and favorable toxicity profiles. Recent studies support earlier integration of immunotherapy, including neoadjuvant and adjuvant approaches, with meaningful gains in event‐free and disease‐free survival.

**Conclusions:**

Immunotherapy is reshaping the management of HNSCC. Ongoing trials, biomarker refinement, and novel strategies are expected to further expand benefit and personalize treatment.

## 1 Introduction

Head and neck squamous cell carcinoma (HNSCC) is a challenging malignancy, often diagnosed at advanced stages, with historically poor outcomes. The advent of immunotherapy, particularly immune checkpoint inhibitors, has revolutionized the therapeutic landscape for HNSCC, offering renewed hope for many patients. Specifically, PD‐1 inhibitors, such as nivolumab and pembrolizumab, have shown efficacy in recurrent, metastatic, and locally advanced disease. These agents work by preventing cancer cells from turning off the local immune response, allowing the body's own immune system to attack the cancer. This paper provides an in‐depth summary of the recent pivotal clinical trials of immunotherapy in HNSCC (exclusive of nasopharyngeal cancer) that have significant implications for clinical practice. Questions remain about the optimal timing of immunotherapy administration (concurrent vs. sequential, neoadjuvant and/or adjuvant), the role of predictive biomarkers, and how best to integrate immunotherapy with existing standard of care modalities. Ongoing preclinical and clinical studies will be required to address these questions. Further, other forms of immunotherapy, including tumor vaccines, adoptive immunotherapy, and cell‐based therapies, require ongoing clinical investigation.

## 2 Rationale of Immunotherapy

Cancer immunotherapy has redefined the therapeutic landscape by demonstrating that durable tumor control can be achieved through restoration of endogenous antitumor immune responses rather than direct cytotoxicity. Although malignant cells express neoantigens that are potentially immunogenic, tumor progression is largely enabled by immune tolerance and active suppression within the tumor microenvironment, mediated by regulatory immune cells, inhibitory cytokines, and immune checkpoint pathways that restrain effector T‐cell function [1]. Conceptual advances in tumor immunology have established immune checkpoints as dominant regulators of antitumor immunity and revealed that their blockade can reverse T‐cell exhaustion, reprogram the tumor–immune equilibrium, and generate long‐lasting clinical benefit [2, 3].

Topalian et al. conducted the first Phase 1 trial of nivolumab (BMS‐936558), a fully human monoclonal antibody that blocks the programmed death 1 (PD‐1) inhibitory receptor on T cells [4]. In 296 heavily pretreated patients with advanced solid tumors, the drug demonstrated significant antitumor activity, with cumulative objective response rates (ORRs) of 28% for melanoma, 27% for renal‐cell cancer, and 18% for non‐small‐cell lung cancer. Most responses were durable, with 65% of those followed for at least 1 year maintaining their response. While a maximum tolerated dose was not reached, Grade 3 or 4 treatment‐related adverse events occurred in 14% of patients, including three deaths from pulmonary toxicity (pneumonitis). Furthermore, preliminary analysis revealed a strong correlation between PD‐L1 expression on tumor cells and clinical response: 36% of patients with PD‐L1‐positive tumors achieved an objective response, whereas none with PD‐L1‐negative tumors did, suggesting that PD‐L1 expression may serve as a predictive biomarker for therapeutic benefit [4].

The success of PD‐1 blockade across multiple tumor types established a new paradigm for cancer treatment and prompted investigation of this approach in additional malignancies characterized by immune evasion. In HNSCC, a disease marked by profound immune dysregulation and frequent expression of programmed death‐ligand 1 (PD‐L1) on both tumor and infiltrating immune cells, engagement of the PD‐1/PD‐L1 axis represents a critical mechanism of immune escape. Clinical translation of this biology has been validated by multiple successful Phase III trials demonstrating that PD‐1 blockade with pembrolizumab yields clinically meaningful and durable responses in patients with recurrent or metastatic (R/M) HNSCC, including biomarker‐unselected populations, thereby establishing immune checkpoint inhibition as a cornerstone of systemic therapy in this disease [5]. Table 1 summarizes the Clinical Trials of Immunotherapy in HNSCC.

**TABLE 1:** Clinical trials of immunotherapy in head and neck squamous cell carcinoma.

Trial name	Phase	Population	Intervention	Primary endpoint	Key results
KEYNOTE‐012	Ib	Recurrent/metastatic HNSCC (*n* = 192)	Pembrolizumab monotherapy	ORR, OS, safety	ORR 18%; median OS 8 months; median DOR > 50 weeks; Grade 3–4 AEs < 10%
KEYNOTE‐040	III	Platinum‐refractory R/M HNSCC	Pembrolizumab vs. standard chemotherapy (MTX/docetaxel/cetuximab)	OS, PFS	Median OS 8.4 vs. 6.9 months (HR 0.80, 95% CI 0.65–0.98); ORR 14.6%; Grade 3+ AEs 13% vs. 36%
KEYNOTE‐048	III	Previously untreated R/M HNSCC	Pembrolizumab ± chemotherapy vs. cetuximab + chemotherapy	OS, PFS	Pembrolizumab alone: OS improved in PD‐L1 CPS ≥ 1 (12.3 vs. 10.3 months, HR 0.78) and CPS ≥ 20 (14.9 vs. 10.7 months, HR 0.61); Pembrolizumab + chemo: OS 13.0 vs. 10.7 months (HR 0.77)
CheckMate 141	III	Platinum‐refractory R/M HNSCC	Nivolumab vs. investigator's choice chemotherapy	OS	Median OS 7.5 vs. 5.1 months (HR 0.70, 95% CI 0.51–0.96); 1‐year OS 36% vs. 16%; 2‐year OS 16.9% vs. 6%; Grade 3–4 AEs 13% vs. 35%
CheckMate 651	III	First‐line R/M SCCHN	Nivolumab + ipilimumab vs. EXTREME regimen	OS	Did not meet primary endpoint for OS; median DOR 32.6 vs. 7.0 months in high CPS patients; better safety profile than EXTREME
CheckMate 714	II	Platinum‐refractory and platinum‐eligible R/M SCCHN	Nivolumab + ipilimumab vs. nivolumab monotherapy	ORR	Did not meet primary endpoint; ORR 13.2% (combo) vs. 18.3% (mono) in platinum‐refractory; no OS/PFS benefit for combination
HAWK	II	Platinum‐refractory R/M SCCHN with PD‐L1 ≥ 25%	Durvalumab monotherapy	ORR, OS	ORR 16.2%; median OS 7.1 months; HPV+ patients: ORR 29.4%, OS 10.2 months
CONDOR	II	R/M SCCHN with PD‐L1 low/negative	Durvalumab + tremelimumab vs. durvalumab or tremelimumab monotherapy	ORR	ORR 7.8% (combo) vs. 9.2% (durvalumab); Grade 3/4 immune AEs 6% in combination arm; manageable toxicity
EAGLE	III	Platinum‐refractory R/M SCCHN	Durvalumab ± tremelimumab vs. standard chemotherapy	OS	No significant OS improvement; higher survival rates at 12–24 months with durvalumab; durable responses in complete remission patients
KESTREL	III	First‐line R/M SCCHN	Durvalumab ± tremelimumab vs. EXTREME regimen	OS in PD‐L1 high	Failed to show OS superiority; median OS 10.9 months in both groups; 24.3% of chemo arm received subsequent immunotherapy
GORTEC 2018–01 (NIVOPOST‐OP)	III	High‐risk resected LA‐HNSCC with ECS, +margins, > 4 nodes, or PNI	Nivolumab + cisplatin‐RT + maintenance nivolumab vs. cisplatin‐RT alone	DFS	3‐year DFS 63.1% vs. 52.5% (HR 0.76, 95% CI 0.60–0.98, *p* = 0.034); 24% relative risk reduction; benefit independent of PD‐L1
KEYNOTE‐689	III	Locally advanced HNSCC	Neoadjuvant/adjuvant pembrolizumab + surgery ± adjuvant RT/CRT vs. standard care	EFS	36‐month EFS 57.6% vs. 46.4% (HR 0.73, 95% CI 0.58–0.92, *p* = 0.008); benefit regardless of PD‐L1 CPS; no unexpected toxicities
JAVELIN Head and Neck 100	III	Previously untreated high‐risk LA‐SCCHN	Avelumab + CRT + maintenance vs. placebo + CRT	PFS, OS	No improvement in PFS or OS in general population; exploratory analysis suggested benefit in PD‐L1 TPS > 25%
PembroRad (GORTEC 2015–01)	II	LA‐SCCHN unfit for high‐dose cisplatin	Pembrolizumab + RT vs. cetuximab + RT	Locoregional control at 15 months	Did not meet primary endpoint; LRC 60% vs. 59%; significantly lower toxicity with pembrolizumab (fewer Grade ≥ 3 mucositis and radiodermatitis)
REWRITE (GORTEC 2018–02)	II	Frail patients ineligible for standard CRT	Durvalumab + reduced‐volume RT (primary site only, limited PNI)	Regional nodal control	96.4% regional nodal control at 1 year in non‐irradiated neck; feasible with limited N0 irradiation
REACH (GORTEC 2017–01)	III	LA‐SCCHN (fit and unfit patients)	Avelumab + cetuximab‐RT vs. cisplatin‐CRT (fit) or cetuximab‐RT (unfit)	Safety, efficacy	Safety phase (*n* = 41): 12% Grade IV acute AEs; tolerable combination; no treatment‐related deaths during concurrent phase
IMvoke010	III	High‐risk LA‐SCCHN after definitive treatment	Atezolizumab maintenance (1 year) vs. placebo	EFS, OS	Failed to improve EFS or OS; well‐tolerated; no new safety signals
NRG‐HN004	II/III	Cisplatin‐contraindicated patients	Durvalumab + RT vs. cetuximab + RT	PFS, OS	Stopped early for futility; Phase II 2‐year PFS: 50.6% (durvalumab) vs. 63.7% (cetuximab); cetuximab showed one of highest control rates in this setting
IMCISION	Ib/IIa	Resectable LA‐HNSCC	Neoadjuvant nivolumab vs. nivolumab + ipilimumab before surgery	MPR, feasibility	MPR 35% (combo) vs. 17% (mono); no tumor relapse in MPR patients at 24‐month median follow‐up; feasible and efficacious
KEYNOTE‐412	III	Unresected high‐risk LA‐SCCHN	Pembrolizumab + CRT + maintenance vs. placebo + CRT	EFS	Initial analysis did not show statistical significance; long‐term follow‐up (median 74.4 months): EFS HR 0.79; particularly notable benefit in PD‐L1 CPS ≥ 1

## 3 Immunotherapy in Recurrent and/or Metastatic HNSCC

### 3.1 KEYNOTE‐012

The KEYNOTE‐012 trial was an early‐phase (Phase Ib) multicenter study evaluating pembrolizumab, which blocks the PD‐1 protein on T cells, in patients with R/M HNSCC. Among 192 treated patients, the ORR was 18%, with some responses lasting over a year [6]. Median overall survival (OS) was 8 months, and the median duration of response exceeded 50 weeks. Pembrolizumab was generally well tolerated, with Grade 3–4 treatment‐related adverse events observed in less than 10% of patients. Importantly, responses were seen in both HPV‐positive and HPV‐negative tumors, suggesting broad applicability [7].

### 3.2 KEYNOTE‐040

KEYNOTE‐040 was a Phase III trial comparing pembrolizumab to standard single‐agent chemotherapy (methotrexate, docetaxel, or cetuximab) in recurrent/metastatic HNSCC post platinum‐failure [8]. Pembrolizumab improved median OS (8.4 vs. 6.9 months; HR 0.80, 95% CI 0.65–0.98) and demonstrated a favorable safety profile, with fewer severe treatment‐related adverse events (13% vs. 36%). These findings reinforced pembrolizumab as a standard of care in the relapsed/refractory setting [8].

### 3.3 KEYNOTE‐048

In the KEYNOTE‐048 Phase III trial, pembrolizumab was evaluated as monotherapy and in combination with chemotherapy versus cetuximab plus chemotherapy in untreated locally incurable R/M HNSCC. Pembrolizumab monotherapy improved OS in patients with a PD‐L1 combined positive score (CPS) ≥ 1 (12.3 vs. 10.3 months; HR 0.78) and CPS ≥ 20 (14.9 vs. 10.7 months; HR 0.61) [9]. In the overall study population, pembrolizumab plus chemotherapy also prolonged OS versus cetuximab with chemotherapy (13.0 vs. 10.7 months; HR 0.77). The toxicity profile favored pembrolizumab monotherapy, with lower rates of grade ≥ 3 adverse events compared to the chemotherapy regimen [9]. The results of this clinical trial suggest that pembrolizumab monotherapy is an appropriate, FDA approved, NCCN‐recommended first‐line treatment for PD‐L1‐positive R/M HNSCC.

### 3.4 CheckMate 141

CheckMate 141 was a multicenter, randomized Phase III trial comparing nivolumab, a PD‐1 inhibitor, to investigator's choice of standard therapy in patients with platinum‐refractory recurrent/metastatic HNSCC. Nivolumab significantly improved median OS (7.5 vs. 5.1 months; HR 0.70, 95% CI 0.51–0.96) and 1‐year OS rates (36% vs. 16%). The drug was associated with durable responses and fewer Grade 3–4 adverse events (13% with nivolumab vs. 35% in the standard therapy group) [10]. With 2‐year follow‐up, nivolumab continues to show a survival advantage (HR of 0.68) and the 24‐month OS rate was 16.9% versus 6% with standard chemotherapy [11]. Benefit was seen regardless of PD‐L1 expression level or HPV status. Further, nivolumab stabilized or improved patient‐reported quality of life compared with standard chemotherapy. CheckMate 141 was the first Phase 3 trial to show a statistically significant OS benefit with a PD‐1 inhibitor in a platinum‐refractory, recurrent/metastatic HNSCC setting, establishing nivolumab as a new standard of care in this setting.

### 3.5 CheckMate 651

CheckMate 651 was a Phase III trial comparing first‐line nivolumab plus ipilimumab to the EXTREME regimen in platinum‐eligible R/M SCCHN. Ipilimumab targets CTLA‐4. The trial did not meet its primary endpoint of improved OS in the overall population or the PD‐L1 CPS 20 population. Despite this, dual immunotherapy demonstrated remarkably durable responses (median 32.6 vs. 7.0 months for EXTREME in high CPS patients) and a significantly better safety profile than the chemotherapy triplet [12].

### 3.6 CheckMate 714

This double‐blind Phase II trial evaluated the clinical benefit of adding ipilimumab to nivolumab versus nivolumab alone as first‐line treatment for both platinum‐refractory and platinum‐eligible R/M SCCHN. The study did not meet its primary endpoint of ORR benefit in the platinum‐refractory population (13.2% for combo vs. 18.3% for mono). No OS or PFS benefit was found for the combination over nivolumab monotherapy in either population, although the safety profile remained manageable [13].

### 3.7 HAWK

The HAWK trial was an international, single‐arm Phase II study assessing durvalumab monotherapy in platinum‐refractory R/M SCCHN patients with high PD‐L1 expression (≥ 25% tumor cells) [14]. It reported an ORR of 16.2% and a median OS of 7.1 months. An exploratory analysis found that HPV‐positive patients had a numerically higher response rate (29.4%) and longer survival (10.2 months) compared to HPV‐negative patients.

### 3.8 CONDOR

The CONDOR trial was a Phase II randomized study testing durvalumab plus tremelimumab or monotherapies in R/M SCCHN patients with PD‐L1‐low/negative disease. The addition of the CTLA‐4 inhibitor tremelimumab did not appear to improve outcomes over durvalumab alone, with ORRs of 7.8% and 9.2%, respectively. All three treatment regimens exhibited a manageable toxicity profile, with Grade 3/4 immune‐mediated adverse events occurring in only 6% of the combination arm [15].

### 3.9 EAGLE

The EAGLE trial was a Phase III study comparing durvalumab monotherapy or durvalumab plus tremelimumab to investigator's choice standard‐of‐care chemotherapy in patients with platinum‐refractory R/M SCCHN. Durvalumab is a monoclonal antibody that targets PD‐L1 while tremelimumab targets CTLA‐4. The trial did not demonstrate a statistically significant improvement in OS for either durvalumab arm versus chemotherapy. However, durvalumab showed clinical activity and higher survival rates at 12 to 24 months compared to chemotherapy, with a more durable response in patients who reached complete remission [16].

### 3.10 KESTREL

KESTREL was a global Phase III trial evaluating first‐line durvalumab (with or without tremelimumab) versus the standard EXTREME regimen (platinum, 5‐FU, and cetuximab) in R/M SCCHN [17]. The trial failed to show OS superiority for durvalumab monotherapy in patients with high PD‐L1 expression, yielding a median OS of 10.9 months in both groups. A likely confounding factor was the high rate of subsequent immunotherapy (24.3%) received by patients in the chemotherapy arm after their initial treatment failed.

## 4 Immunotherapy in Newly Diagnosed, Previously Untreated HNSCC

### 4.1 GORTEC 2018‐01 NIVOPOST‐OP

GORTEC 2018–01 (NIVOPOST‐OP) is a Phase III randomized controlled trial in patients with completely resected locally advanced HNSCC with high‐risk pathologic features after surgery (e.g., extracapsular nodal extension, microscopically positive margins, multiple ≥ 4 cervical lymph nodes involved, perineural invasion) [18]. The experimental arm included lead‐in nivolumab plus concurrent nivolumab with adjuvant cisplatin‐radiation (chemoradiotherapy [CRT]) plus adjuvant maintenance nivolumab every 4 weeks for 6 cycles. The primary endpoint was disease‐free survival (DFS, time to relapse or death). The results demonstrated improved 3‐year DFS in the nivolumab arm as compared to the control arm (63.1% vs. 52.5%) with a hazard ratio of 0.76 (95% CI, 0.60–0.98) and a *p* value of 0.034. This result represents a 24% relative reduction in risk of recurrence or death is nivolumab plus CRT versus CRT alone. In this study, the benefits appear to be independent of PD‐LI expression.

### 4.2 KEYNOTE‐689

KEYNOTE‐689 is a groundbreaking Phase III multicenter, randomized clinical trial evaluating neoadjuvant and adjuvant pembrolizumab in combination with standard care (surgery and adjuvant radiotherapy with or without cisplatin) in locally advanced HNSCC to standard care alone (control group). In the first interim analysis, the addition of neoadjuvant and adjuvant pembrolizumab to standard care significantly improved event‐free survival (EFS) among participants with locally advanced HNSCC. This trial investigated immunotherapy in curative‐intent settings and with an intent to redefine standards for locally advanced disease [19]. The 36‐month EFS in all participants, regardless of PD‐L1 CPS, was 57.6% as compared to 46.4% in the control arm with a hazard ratio for progression, recurrence, or death of 0.73 (95% CI: 0.58–0.92, *p* = 0.008, two‐sided) [20]. Adverse events were consistent with prior immunotherapy studies and did not reveal unexpected toxicities, affirming the safety and efficacy of adding immunotherapy to curative‐intent regimens [20]. This landmark study validates the role neoadjuvant‐adjuvant immunotherapy plays in previously untreated, locally advanced HNSCC. In the first interim analysis, OS was not significant. Longer follow‐up is needed to determine if OS data will reach statistical significance.

### 4.3 JAVELIN Head and Neck 100

This Phase III trial evaluated the efficacy of avelumab in combination with concurrent CRT and maintenance versus placebo plus CRT for previously untreated high‐risk locally advanced squamous cell carcinoma of the head and neck (LA‐SCCHN). The study primarily found that the addition of the PD‐L1 inhibitor to standard‐of‐care CRT did not improve progression‐free survival (PFS) or OS in the general population. However, exploratory results suggested avelumab might offer a benefit in patients with higher PD‐L1 expression (tumor proportion score > 25%) [21].

### 4.4 PembroRad (GORTEC 2015‐01)

PembroRad was a multicenter Phase II trial comparing pembrolizumab plus radiotherapy (RT) to the standard‐of‐care cetuximab plus RT in patients with LA‐SCCHN who were unfit for high‐dose cisplatin. The trial did not meet its primary endpoint of improved locoregional control (LRC) at 15 months, as both arms showed similar control rates (60% vs. 59%). Notably, the pembrolizumab arm was associated with significantly lower toxicity, specifically fewer cases of Grade 3 or higher mucositis and radiodermatitis [22].

### 4.5 Rewrite (GORTEC 2018‐02)

REWRITE was a single‐arm Phase II trial evaluating the efficacy of durvalumab combined with RT restricted to the primary tumor site and involved lymph nodes, utilizing reduced‐volume prophylactic neck irradiation (PNI) of N0 regions, in frail patients ineligible for standard CRT. The study met its primary endpoint, demonstrating a 96.4% regional nodal control rate at one year in the non‐irradiated neck. These results suggest that very limited N0 irradiation is feasible and does not compromise nodal control in this specific subset of patients [23].

### 4.6 Reach (GORTEC 2017‐01)

This Phase III trial investigated adding avelumab to a cetuximab‐RT backbone compared to standard cisplatin‐based CRT (in fit patients) or cetuximab‐RT alone (in unfit patients). The results of the safety phase involving the first 41 experimental patients indicated that the combination was tolerable, with a 12% rate of Grade IV acute adverse events, which was similar to historical standard rates. No treatment‐related deaths occurred in the experimental arms during the concurrent phase [24]. In cisplatin‐unfit patients, a favorable effect of adding avelumab to cetuximab was seen on PFS, local‐regional control, and distant metastasis, but the primary endpoint on PFS was not met. In cisplatin‐fit patients, the futility boundary for efficacy was crossed, favoring standard‐of‐care cisplatin [25].

### 4.7 IMvoke010

IMvoke010 was a global, Phase III, double‐blind trial that assessed atezolizumab maintenance therapy for 1 year following definitive multimodal treatment (surgery, radiation, or chemotherapy) in high‐risk LA‐SCCHN patients. The study failed to improve investigator‐assessed EFS or OS compared to placebo. Despite the lack of clinical benefit, atezolizumab was generally well‐tolerated, with no new or unexpected safety signals reported [26].

### 4.8 NRG‐HN004

This Phase II/III trial compared durvalumab plus RT to cetuximab plus RT in patients for whom cisplatin was contraindicated due to comorbidities or age. The trial was stopped early for futility after an interim analysis showed durvalumab was unlikely to improve outcomes over cetuximab. Final Phase II results showed a numerically lower 2‐year PFS for durvalumab (50.6%) compared to cetuximab (63.7%), which represented one of the highest control rates reported for the cetuximab arm in this setting [27].

### 4.9 IMCISION

IMCISION was a Phase Ib/IIa trial evaluating the feasibility and pathological efficacy of neoadjuvant nivolumab (monotherapy) or nivolumab plus ipilimumab (dual therapy) administered prior to surgery. The trial induced a major pathological response (MPR) in 35% of combo‐treated patients and 17% of monotherapy‐treated patients. Notably, none of the patients who achieved an MPR experienced a tumor relapse during a median follow‐up of 24 months, indicating that neoadjuvant combination therapy is both feasible and encouragingly efficacious [28].

### 4.10 KEYNOTE‐412

This randomized Phase III trial compared pembrolizumab plus concurrent CRT followed by maintenance versus placebo plus CRT in unresected high‐risk LA‐SCCHN. While initial analyses did not find statistical significance, long‐term follow‐up (median 74.4 months) demonstrated a clinically meaningful EFS benefit for the pembrolizumab group versus placebo (HR 0.79, 95% CI = 0.65–0.96). Efficacy was particularly notable in patients with PD‐L1 CPS > 1, where median EFS was significantly longer compared to the placebo group (HR = 0.80, 95% CI = 0.64–0.98) [29].

## 5 Other Immunotherapeutic Approaches and Future Directions

Therapeutic cancer vaccines, adoptive cellular therapies, and cytokine‐based treatment modalities have been explored in early‐phase clinical trials. While objective responses are infrequent, some patients demonstrate immune activation and prolonged disease stabilization, indicating the potential for further development and combination strategies [30].

Antibody‐drug conjugates (ADCs), often described as “biological missiles,” represent a transformative class of targeted cancer therapy designed to improve the therapeutic window by delivering potent cytotoxic payloads directly to tumor cells. These agents are composed of three critical components: a monoclonal antibody that targets specific tumor antigens, a chemical linker, and a highly toxic payload. Once the antibody binds to its target on the cancer cell surface, the entire complex is internalized and transported to lysosomes, where the linker is cleaved to release the drug, leading to cell death. This targeted mechanism aims to minimize off‐target exposure to healthy tissues, thereby reducing the systemic toxicity often associated with conventional chemotherapy [31, 32, 33].

In the specific context of HNSCC, ADCs are emerging as a viable alternative for patients with R/M disease who have limited treatment options following standard platinum‐based chemotherapy and immunotherapy [34]. Current research is investigating various promising targets for HNSCC, including established antigens like EGFR, HER2, and Trop‐2, as well as novel targets such as SLC3A2 and CEACAM5. For example, the ADC 19G4‐MMAE, which targets SLC3A2, has shown significant antitumor efficacy in preclinical HNSCC models with a favorable safety profile [35]. Despite these advancements, successful clinical integration faces challenges such as antigen heterogeneity and the development of adaptive resistance, which current studies are attempting to overcome through innovative ADC structures and combination strategies.

### 5.1 Safety and Adverse Events

Immunotherapy in HNSCC is generally well tolerated. Immune‐mediated adverse events such as hypothyroidism, pneumonitis, colitis, and skin reactions are most common, with Grade 3–4 events occurring in 10%–20% of patients. Early recognition and management allow for mitigation of serious risks, making immunotherapy a viable option in this population [36].

### 5.2 Integrating Immunotherapy With Surgery, Chemotherapy and Radiation

The role both radiotherapy and chemotherapy may play in enhancing immunotherapeutic responses is under active investigation [30]. Better outcomes have been demonstrated in the definitive treatment of HNSCC with CRT when pembrolizumab has been delivered sequentially rather than concurrently [37]. The groundbreaking Phase 3 KEYNOTE‐689 trial does demonstrate improved EFS rates with neoadjuvant/adjuvant delivery of pembrolizumab [38]. In further support of sequential and perioperative immune checkpoint immunotherapy, a recent landmark Phase 2 clinical trial in resectable Stage III or IV melanoma has demonstrated improved survival with neoadjuvant‐adjuvant pembrolizumab over adjuvant therapy alone [39]. EFS was 72% (95% confidence interval [CI], 64–80) in the neoadjuvant‐adjuvant group and 49% (95% CI, 41–59) in the adjuvant‐only group. Taken together, these studies strongly support the further study of neoadjuvant‐adjuvant immune checkpoint inhibition in the perioperative setting.

## 6 Conclusions

The collective data from these pivotal trials have established immune checkpoint inhibitors, specifically pembrolizumab and nivolumab, as new standards of care for recurrent and metastatic HNSCC, especially in populations with high PD‐L1 expression or platinum‐refractory disease. Incorporation into first‐line and curative‐intent regimens is being validated by ongoing Phase III studies, with the most recent evidence (e.g., GORTEC 2018–91 (NIVOPOST‐OP) and KEYNOTE‐689) supporting earlier use and in combination with standard care in the neoadjuvant/adjuvant setting. Immunotherapy has transformed the prognosis for many patients, offering improved survival, durable responses, and generally manageable toxicity. Continued biomarker‐driven research and novel immunotherapeutic strategies are essential to expand benefit to a larger proportion of patients and further personalize therapy to improve outcomes.

## Ethics Statement

The authors have nothing to report.

## Data Availability

Data sharing not applicable to this article as no datasets were generated or analyzed during the current study.
